# Early identification of bleeding in trauma patients: external validation of traumatic bleeding scores in the Swiss Trauma Registry

**DOI:** 10.1186/s13054-022-04178-8

**Published:** 2022-09-28

**Authors:** Alan Costa, Pierre-Nicolas Carron, Tobias Zingg, Ian Roberts, François-Xavier Ageron, for the Swiss Trauma Registry

**Affiliations:** 1 Department of Emergency Medicine, Lausanne University Hospital, University of Lausanne, Rue du Bugnon 46, 1011 Lausanne, Switzerland; 2 Department of Surgery, Lausanne University Hospital, University of Lausanne, Lausanne, Switzerland; 3 Clinical Trials Unit, London School of Hygiene and Tropical Medicine, London, WC1E 7HT UK; 4 Swiss Trauma Board, Lausanne, Switzerland

**Keywords:** Haemorrhage, Trauma, Massive transfusion, Death from bleeding, Score, Prognostic model

## Abstract

**Background:**

Early identification of bleeding at the scene of an injury is important for triage and timely treatment of injured patients and transport to an appropriate facility. The aim of the study is to compare the performance of different bleeding scores.

**Methods:**

We examined data from the Swiss Trauma Registry for the years 2015–2019. The Swiss Trauma Registry includes patients with major trauma (injury severity score (ISS) ≥ 16 and/or abbreviated injury scale (AIS) head ≥ 3) admitted to any level-one trauma centre in Switzerland. We evaluated ABC, TASH and Shock index (SI) scores, used to predict massive transfusion (MT) and the BATT score and used to predict death from bleeding. We evaluated the scores when used prehospital and in-hospital in terms of discrimination (C-Statistic) and calibration (calibration slope). The outcomes were early death within 24 h and the receipt of massive transfusion (≥ 10 Red Blood cells (RBC) units in the first 24 h or ≥ 3 RBC units in the first hour).

**Results:**

We examined data from 13,222 major trauma patients. There were 1,533 (12%) deaths from any cause, 530 (4%) early deaths within 24 h, and 523 (4%) patients who received a MT (≥ 3 RBC within the first hour). In the prehospital setting, the BATT score had the highest discrimination for early death (C-statistic: 0.86, 95% CI 0.84–0.87) compared to the ABC score (0.63, 95% CI 0.60–0.65) and SI (0.53, 95% CI 0.50–0.56), *P* < 0.001. At hospital admission, the TASH score had the highest discrimination for MT (0.80, 95% CI 0.78–0.82). The positive likelihood ratio for early death were superior to 5 for BATT, ABC and TASH. The negative likelihood ratio for early death was below 0.1 only for the BATT score.

**Conclusions:**

The BATT score accurately estimates the risk of early death with excellent performance, low undertriage, and can be used for prehospital treatment decision-making. Scores predicting MT presented a high undertriage rate. The outcome MT seems not appropriate to stratify the risk of life-threatening bleeding.

*Trial registration*: Clinicaltrials.gov, NCT04561050. Registered 15 September 2020.

**Supplementary Information:**

The online version contains supplementary material available at 10.1186/s13054-022-04178-8.

## Introduction

Trauma is a leading cause of death worldwide [1], and bleeding is one of the most preventable causes of traumatic death [2–5]. Early identification of bleeding at the scene of injury is important for triage and timely treatment of injured patients, for ensuring that patients are taken to the most appropriate facility and for trauma team activation [6].

Accurate prehospital prediction of the risk of life-threatening bleeding and the need for blood transfusion could improve patient outcome. Many trauma scores predict death from any cause after injury but far fewer predict bleeding related outcomes. Most of those predict surrogates of bleeding such as massive transfusion (MT) or the use of specific interventions for haemorrhage [7, 8].

MT is defined as administration of ≥ 10 units of red cells in the first 24 h after injury or ≥ 3 units in the first hour after injury [6–13]. The most popular trauma scores used to assess traumatic bleeding are the TASH [14, 15] and ABC score [16, 17]. Both predict MT as a surrogate of risk of death from bleeding and include clinical parameters as well as imaging and laboratory values. Because they involve imaging and laboratory testing these scores cannot be readily assessed at the scene. Shock index (SI) [18] has recently attracted attention because it can be used easily at the scene of the injury [19].

The BATT score is a new prognostic model that can be used in the pre-hospital setting to predict death from bleeding. The score was developed in a large international cohort [20] of trauma patients and externally validated using data from England and Wales [21].

We used data from the Swiss Trauma Registry to externally validate existing prognostic scores for traumatic bleeding when used prehospital and after hospital admission. We compared score performances in terms of overall performance, discrimination and calibration [22].

## Methodology

We compared the performance of different bleeding scores using data from the Swiss Trauma Registry (STR) from January 1, 2015, to December 31, 2019. The STR includes patients with major trauma (injury severity score (ISS) ≥ 16 and/or abbreviated injury scale (AIS) head ≥ 3) admitted to any of twelve level-one trauma centres in Switzerland. We excluded patients with isolated burns (including electric shock) or if the burn was the first injury, patients arriving in hospital without sign of life where no diagnostic or therapeutic measures had been initiated, patients with choking or hanging without any other injury, and victims of drowning.

### Calculation of bleeding scores

We collected a set of demographic data, first prehospital and in-hospital physiological variables (Heart Rate [HR], systolic blood pressure [SBP], respiratory rate [RR], peripheral capillary oxygen saturation [SpO2], Glasgow coma scale [GCS]), first measures of in-hospital biochemical values (haemoglobin [Hb], base excess [BE]), first-read imaging (Focused Assessment with Sonography for Trauma [FAST]) and blood transfusion records (Type of Blood product, volume and time). We evaluated the most widely used scores for predicting MT (ABC, TASH and SI) and a score that predicts death from bleeding (BATT).

*Assessment of Blood Consumption* (ABC) includes penetrating trauma, SBP, HR and FAST. *Trauma-Associated Severe Haemorrhage* (TASH) includes sex, HR, SBP, Hb, BE, intra-abdominal fluid and complex fracture of the pelvis and/or long bone. *Shock Index* (SI) is defined by the ratio of HR to SBP. *Bleeding Audit for Trauma & Triage* (BATT) includes age, mechanism of trauma (penetrating/high energy), SBP, HR, GCS, RR or SpO2. Details about development and validation of each score are summarized in supplement 1. Others variables collected followed the Utstein-style for major trauma template [23] and regularly cross-checked for external validity and completeness by the register.

### Outcome measures

We assessed the accuracy of the scores to predict two bleeding-related outcomes: (1) death within 24 h of injury and (2) the receipt of a massive transfusion. Because ‘death from bleeding’ was not routinely recorded in the trauma registry, we used death within 24 h of injury, since studies have shown that most bleeding deaths are on the day of the injury. Two studies, one from North America and one based on two large European trauma registries (UK and Germany), show that most deaths from bleeding occur within 24 h of injury, with a peak around 6 h after admission. Head injury deaths occur later, around 72 h after injury [24, 25]. Death is an outcome that matters to patients and is accurately measured. Following an NIH consensus conference, it was recommended that early death can be used as a primary outcome measure in clinical trials in haemorrhage control [26]. We also performed a sensitivity analysis with very early death (death within 12 h of injury). We used two definitions of massive transfusion: receipt of ≥ 10 RBC units in the first 24 h and receipt of ≥ 3 RBC units within the first hour. Although the first definition (≥ 10 RBC units) is often used in the trauma literature, the second definition is believed by some authors to be more accurate and less vulnerable to survival bias [13]. Because most deaths from bleeding are on the day of the injury, with many deaths in the first few hours after injury, some patients with severe bleeding do not survive long enough to receive ≥ 10 RBC units. This definition of massive bleeding will therefore fail to identify many patients with severe bleeding [11]. A second weakness of massive transfusion as an outcome is that it is less patient centred. Massive transfusion is a medical intervention and not a biological effect of severe bleeding.

### Statistical analysis

The statistical analysis plan for the pre-specified analysis is registered at www.clinicaltrials.gov: NCT04561050. The STR has authorized us to access and process the registry data (ID-project: STR-ID 8) and granted us with the permission to publish the manuscript in accordance with the STR publication guidelines. Descriptive statistics included frequencies, 95% confidence interval (CI) for categorical variables and either the mean (SD) or median (Interquartile range [IQR]) for continuous variables, according to data distribution. We compared the overall performance with the Brier score, discrimination and calibration of the different scores for the prediction of MT and early death at scene and at hospital admission. The Brier Scores for the ABC score and SI were not calculated as they are not able to predict the probability of an outcome. For discrimination, we estimated the sensitivity, specificity, positive and negative likelihood ratio for the cut-off point of each score. We plotted the receiving operating characteristic (ROC) curve and estimated the area under the ROC curve (AUROC) that corresponds to the concordance statistic (C-Statistic). Definitions of the statistical terms and indicators are shown in supplement 2. For the calibration, we estimated calibration in the large, the ratio of the predicted and observed number of events (P/O). We plotted the observed and predicted probabilities of MT for the TASH score and haemorrhagic death for the BATT by decile of the score and with local regression based on LOESS algorithm [22]. The calibration of the ABC score and SI could not be assessed as they are not able to predict a probability of MT.

### Grey-zone approach

Because no score can perfectly differentiate trauma patients with severe bleeding (who might die or need massive transfusion) from those without severe bleeding (who will not die and not need massive transfusion), we used a grey-zone approach that identifies a middle ‘inconclusive range’ [27]. To set the upper limit of the inconclusive range, we used a score with high (90%) specificity. Patients with scores above this value have a very high risk of severe bleeding and there would be few patients without severe bleeding. Such a high score might be appropriate for deciding who should get an expensive intervention that although effective, might also cause serious side effects. To set the lower limit of the inconclusive range, we use a score with a high sensitivity. The American College of Surgeons recommends a score that gives 95% sensitivity so that only 5% of severe bleeding cases are missed, although up to 50% of patients without severe bleeding may be included [28]. Such a score might be used when deciding who should get a low-cost intervention with an excellent safety profile. Patients with scores between the upper and lower limits are in the ‘grey zone’ where the discriminatory performance of the scores is insufficient to determine whether or not the patient has severe bleeding.

### Missing data

Because there were missing values for some predictors, we used multiple imputation by chained equations, with 20 imputed datasets, to impute missing values for sex, age, SBP, RR, HR, GCS, Hb, BE, RBC and type of injury (penetrating/blunt).

All analyses were performed using STATA software (version 16.0; Stata Corp, College Station, TX, USA).

## Results

We examined data from 13,222 trauma patients. Their characteristics are shown in Table 1. There were 1,533 (11.6%) deaths from any cause, 530 (4.0%) early deaths, 128 (1.0%) patients received 10 RBC within the first 24 h and 523 (4.0%) patients received 3 RBC within the first hour. The mean ISS of patients who received a MT was 32 [SD 13] compared with 21 [SD 10] for those who did not. Of patients who received a MT, 30% died compared with 11% (95% CI 11–13) of those who did not.

**Table 1:** Characteristics of injured patients

	Missin*g* N (%)	All patients (*N* = 13,222) *N* (%)	< 3 RBC units/1 h (*N* = 12,699) *N* (%)	≥ 3 RBC units/1 h (MT) (*N* = 523) *N* (%)
Age, mean (SD)	0	58 (22)	59 (21)	52 (22)
Male	0	9030 (68)	8677 (68)	353 (68)
Circumstances	156 (1)			
Traffic accident		3914 (30)	3707 (30)	210 (41)
Falls		7143 (55)	6933 (55)	210 (41)
Gunshots and Stabbings		233 (2)	198 (2)	36 (7)
Mechanism				
Penetrating	16 (0.12)	871 (6)	774 (6)	97 (19)
High energy	146 (1)	7397 (57)	7031 (56)	366 (71)
Injury Severity Score (ISS)				
Mean (SD)	6 (0.04)	22 (10)	21 (10)	32 (13)
9–15		3145 (24)	3110 (25)	35 (7)
16–24		5252 (40)	5134 (40)	118 (23)
25–34		3721 (28)	3536 (28)	185 (35)
> 35		1098 (8)	913 (7)	185 (35)
AIS head ≥ 3	0	8959 (68)	8675 (68)	284 (54)
Prehospital SBP, Mean (SD)	4412 (33)	135 (31)	136 (31)	116 (37)
< 90 mmHg		497 (6)	410 (5)	87 (22)
Prehospital HR, Mean (SD)	4172 (31)	86 (22)	86 (21)	97 (31)
Prehospital RR, Mean (SD)	9090 (69)	18 (7)	18 (7)	18 (9)
Prehospital SpO2, Mean (SD)	5486 (41)	95 (7)	95 (6)	91 (13)
< 90%		955 (12)	845 (11)	110 (29)
Prehospital GCS category	3798 (29)			
3–8		1841 (20)	1654 (18)	187 (43)
9–12		1090 (11)	1044 (12)	46 (10)
13–15		6486 (69)	6279 (70)	207 (47)
Hospital mortality	8 (0.06)			
All cause of death at 28 days		1533 (11.6)	1376 (10.8)	157 (30.0)
Early death within 24 h		530 (4.0)	448 (3.5)	82 (15.7)

### Performance of bleeding scores

**Fig. 1:**
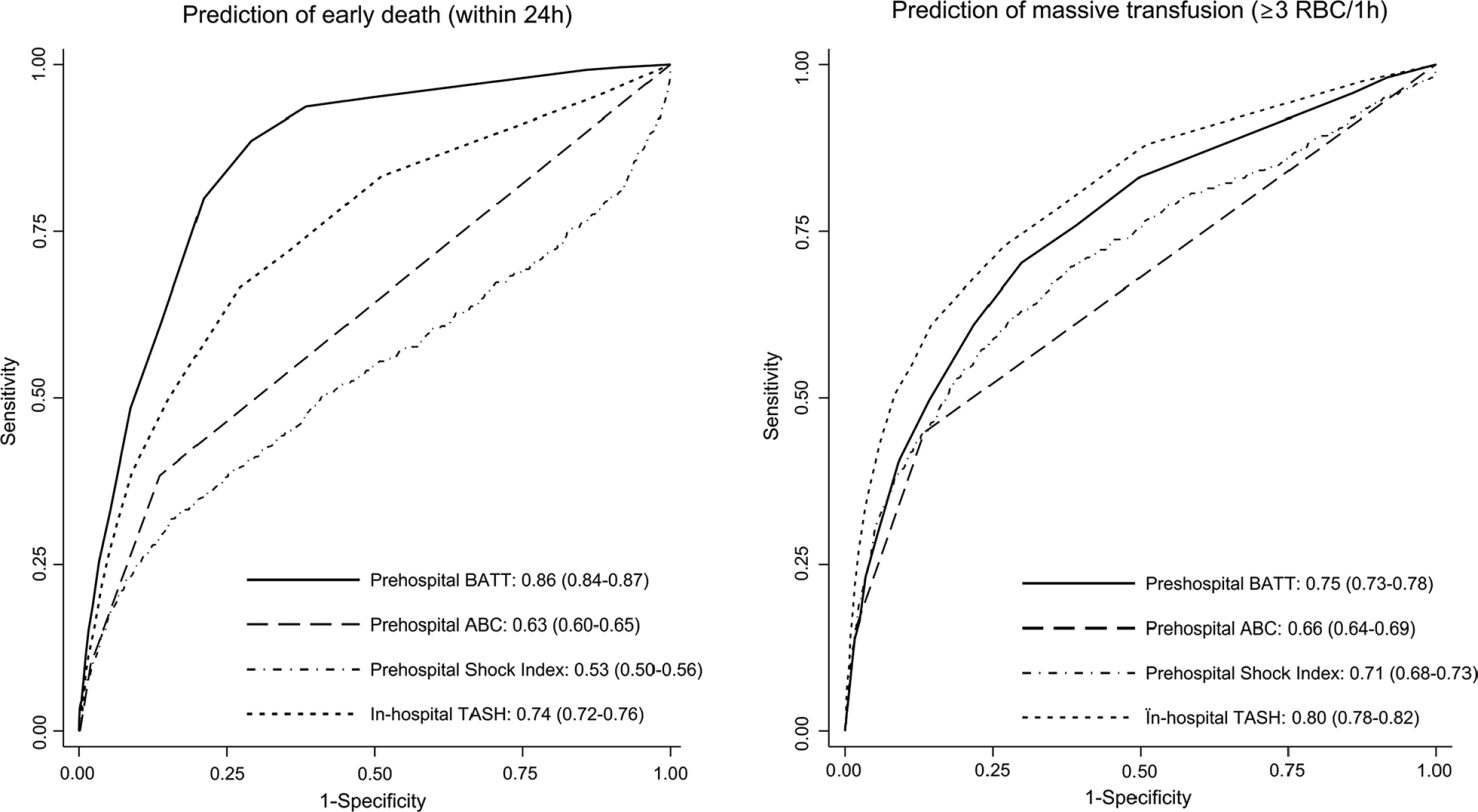
Receiving operating curve (ROC) of bleeding scores

Table 2 shows the performance of bleeding scores calculated pre-hospital and in-hospital. The Brier score for BATT was 0.036 both pre-hospital and in-hospital. The Brier score for the TASH score at hospital admission was 0.060. In the prehospital setting, the BATT score had a higher discrimination for early death than ABC and SI, respectively C-statistic: 0.86, 95% CI (0.84–0.87); 0.63, 95% CI (0.60–0.65); 0.53, 95% CI (0.50–0.56); *P* < 0.001. The BATT score had the highest discrimination for MT in the prehospital setting (C-statistic: 0.75, 95% CI 0.73–0.78) followed by the Shock Index (C-statistic: 0.71, 95% CI 0.68–0.88) and the ABC score (C-statistic: 0.66, 95% CI 0.64–0.69), *P* < 0.01. At hospital admission, the BATT score had the highest discrimination for early death (C-statistic: 0.87, 95% CI 0.86–0.88). The TASH score had the highest discrimination for MT (C-statistic: 0.80, 95% CI 0.78–0.82). Figure 1 shows ROC curves for MT and early death. We presented in supplement 3, ROC curves for very early death (within 12 h) as sensitivity analysis for death from bleeding.

**Table 2:** Performance of bleeding scores

	Overall	Discrimination C-statistic (95% CI)			Calibration				
	Brier score	For early death (within 24 h)	For massive transfusion (3 RBC/1 h)	For massive transfusion (10 RBC/24 h)	Outcome observed % (95% CI)^a^	Outcome predicted % (95% CI)^a^	*P* value	Calibration intercept (95% CI)	Calibration slope (95% CI)
Prehospital									
BATT score	0.036	0.86 (0.84–0.87)*	0.75 (0.73–0.78)*	0.87 (0.84–0.90)	4.0 (3.6–4.3)	2.1 (2.0–2.2)	< 0.001	0.008 (0.005–0.012)	1.48 (1.40–1.56)
Shock Index ^b^	–	0.53 (0.50–0.56)	0.71 (0.68–0.73)	0.84 (0.80–0.88)	–	–		–	–
ABC score ^b,c^	–	0.63 (0.60–0.65)	0.66 (0.64–0.69)	0.82 (0.77–0.86)	–	–		–	–
TASH score^d^	–		–	–	–	–		–	–
In-Hospital									
BATT score	0.034	0.87 (0.86–0.88)*	0.77 (0.75–0.79)	0.89 (0.86–0.91)	4.0 (3.6–4.3)	2.7 (2.6–2.8)	< 0.001	0.005 (0.001–0.008)	1.31 (1.24–1.37)
Shock Index ^b^	–	0.61 (0.58–0.64)	0.74 (0.72–0.77)	0.89 (0.86–0.92)	–	–		–	–
ABC score ^b^	–	0.66 (0.63–0.68)	0.70 (0.67–0.72)	0.84 (0.80–0.87)	–	–		–	–
TASH score	0.060	0.74 (0.72–0.76)	0.80 (0.78–0.82)**	0.94 (0.92–0.95)*	4.0 (3.6–4.3)	6.1 (5.8–6.5)	< 0.001	0.011 (0.008 -0.015)	0.46 (0.44–0.48)

^*^P < 0.001 (compare to the highest score below); **P < 0.01

^a^BATT score predicted death from bleeding compared to early death within 24 h; TASH score predicted massive transfusion (≥ 10 RBC/24 h) compared to ≥ 3 RBC within the first hour

^b^The Brier score, calibration in the large, calibration intercept and calibration slope cannot be estimated as SI and ABC score does not estimate a probability of massive transfusion, early death, or death from bleeding

^c^ABC score was estimated without ultrasonography (FAST) as ultrasonography is not available in routine in the prehospital setting

^d^TASH score in the prehospital setting is not feasible as biological assay and imaging are not available

**Fig. 2:**
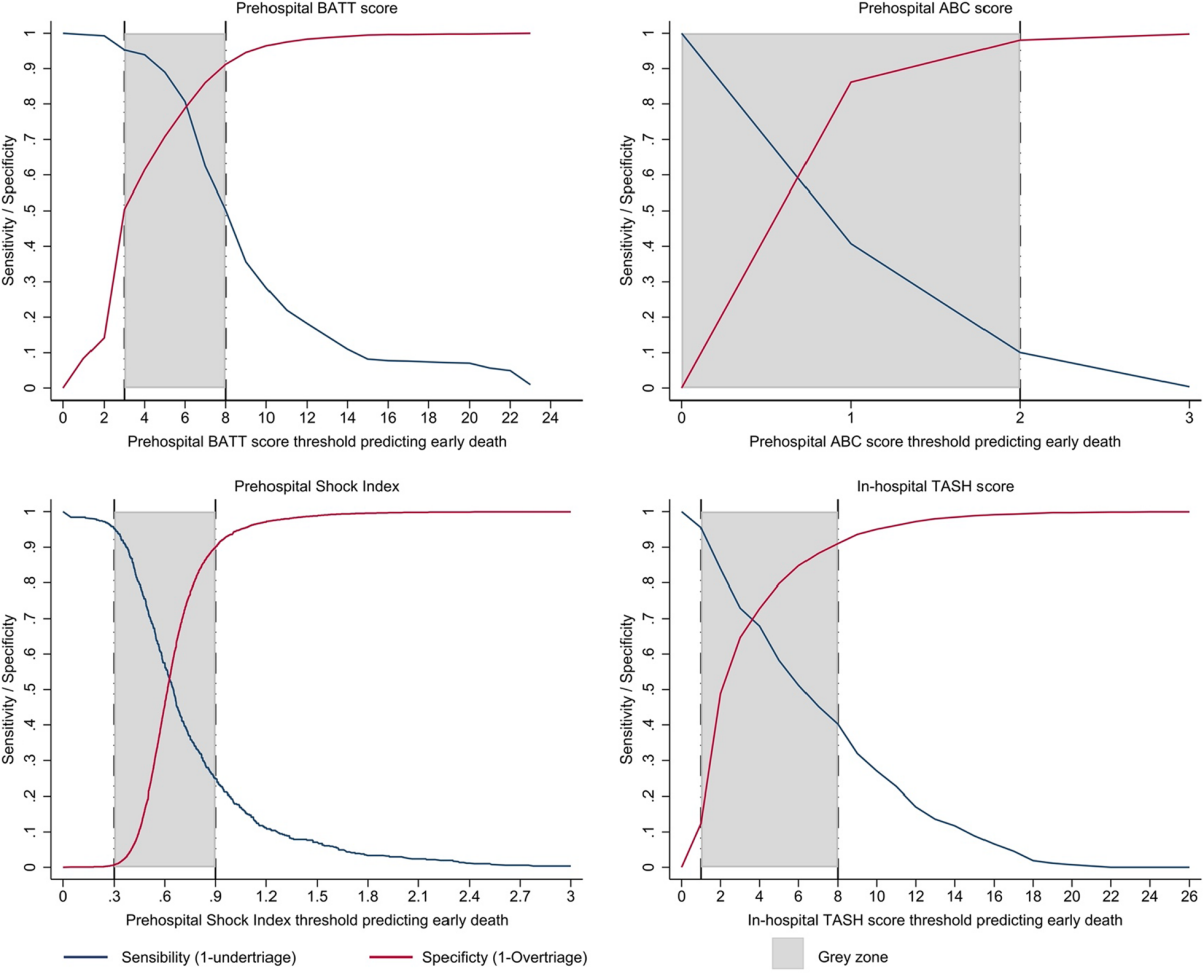
Grey-zone approach with sensitivity and specificity curves for early death prediction

The prehospital BATT score ≥ 3 presented a sensitivity of 95%, a specificity of 50% and a LR+ of 0.09 for early death (Table 3). The sensitivity for early death prediction was low for all thresholds of the ABC score, the TASH score and the SI. ABC score ≥ 2 showed a sensitivity of 10%, which means that 90% of injured dead patients had an ABC score < 2 (Table 3). In the prehospital setting, a BATT score ≥ 8, SI > 0.9 and ABC score ≥ 2 had a specificity of 90% or more for MT and early death (Table 3). At hospital admission, the TASH score ≥ 12 had a high specificity for MT prediction (98%) but a low sensitivity (27%). A prehospital BATT score ≥ 3 showed an undertriage of 5% and an overtriage of 50% for early death (Table 3). Figure 2 summarizes the grey-zone approach. All scores achieved a specificity of 90% for the upper limit. Only the BATT score provides a short grey zone with 5% of under triage and 50 of over triage for the lower limit.

**Table 3:** Discrimination performance by scores threshold

	Sensibility (%)	Undertriage (%)	Specificity (%)	Overtriage (%)	Likelihood ratio +	Likelihood ratio −
Early death (within 24 h)						
Prehospital BATT score						
≥ 3	95.3	4.7	50.5	49.5	1.9	0.09
≥ 8	50.4	49.6	91.1	8.9	5.7	0.54
Prehospital ABC score						
≥ 1	40.7	59.3	86.2	13.8	2.9	0.69
≥ 2	10.0	90.0	98.0	2.0	5.0	0.92
Prehospital Shock Index						
≥ 0.7	42.1		69.2		1.4	0.84
≥ 0.9	25.0	75.0	89.9	10.1	2.5	0.83
In-hospital TASH score						
≥ 1	95.5	4.5	12.4	87.6	1.1	0.37
≥ 8	40.3	59.7	90.1	9.9	4.5	0.66
≥ 12	17.0	83.0	97.2	2.8	6.1	0.85
Massive transfusion (3 RBC/1 h)						
Prehospital BATT score						
≥ 3	83.4	16.6	49.9	50.1	1.7	0.33
≥ 8	40.9	59.1	90.7	9.3	4.4	0.65
Prehospital ABC score						
≥ 1	45.3	54.7	86.3	12.7	3.3	0.63
≥ 2	15.1	84.9	98.2	7.8	8.4	0.86
Prehospital Shock Index						
≥ 0.7	63.1		70.0		2.1	0.53
≥ 0.9	39.4		90.5		4.1	0.67
In-hospital TASH score						
≥ 1	97.5	2.5	12.5	87.5	1.1	0.20
≥ 8	50.9	49.1	91.4	8.6	5.9	0.54
≥ 12	27.3	72.7	97.7	2.3	11.6	0.74

Undertriage = 1- Sensibility; Overtriage = 1 – specificity

For the BATT score, the calibration curve showed slight over-prediction in low-risk patients and under-prediction in intermediate and high-risk patients. For the TASH score at hospital admission, the calibration curve showed over-prediction of MT (Supplement 4).

## Discussion

### Main findings

Only the BATT score accurately predicts the risk of early death in the prehospital setting. The sensitivities of scores predicting MT (ABC, TASH, SI) are low. Their negative likelihood ratio for rule out are too high and make them not suitable for the early identification of life-threatening bleeding. All scores accurately predict MT with a moderate positive likelihood ratio for rule in.

### Strengths and limitations

Our study has important strengths. We validated the scores in data from a large national trauma registry which includes trauma patients with a wide range of bleeding severity. This provided a heterogenous case-mix that allowed for accurate assessment of discrimination [29]. The large number of patients in this study increased the precision of the results. Finally, we used first parameters recorded by paramedics at trauma scene and at hospital admission when the decision of MT had to be made. We used rigorous methods, assessing not only discrimination but all performance criteria, including global performance, discrimination, and calibration. We determined sensitivity and specificity for each threshold and considered the risks of undertriage or overtriage with a grey-zone approach.

Our study also has limitations. Measurement error in predictor variables could affect discrimination and calibration. Random error arises for all predictors (BP, HR, GCS, RR) and leads to reduced discrimination and calibration. Systematic errors arising from the use of monitoring devices are more likely to affect calibration [30]. Because the outcome ‘death from bleeding’ was not available in the STR database, we used early death within 24 h as a proxy [31], subject to misclassification bias due to other cause of death. Massive transfusion is also subject to misclassification due to information bias. Any outcome misclassification would be expected to decrease the C-statistic and reduce the model performance [32] and since the C-statistic was high and model performance was excellent, misclassification is unlikely to be an important weakness for early death. Because MT and some predictors were missing, we imputed theses data. We assumed that data were missing at random. If not, complete case should perform better. We noticed a survival bias on the primary outcome. As some patients may not survive long enough to receive 10 RBC in the first 24 h, MT is subject to misclassification.

### Comparison to other studies

Our patient’s characteristics were similar to other European studies [15, 21]. To the best of our knowledge, the BATT score is the only score that predicts traumatic death from bleeding and could be easily applied at the trauma scene [21]. In our study, we found similar good discrimination to identify MT for TASH, ABC score and SI [14–18, 33, 34]. The MT rate was low and comparable to the lower limit reported in the literature [14–16, 21, 34]. For the TASH score, we observed a clear over-prediction of MT for all risk patients with 6.1% of predicted probability. In the literature, we observed a decrease of MT use over the last years. The German registry reported 14.1% of MT between 1993 and 2003 [14], 8.4% between 2004 and 2007 [15] and 1.7% in between 2015 to 2019 [35]. The decline in MT might be explained by changes in blood management practice in severe trauma. Moreover, early identification of acute trauma coagulopathy by thromboelastography might have decreased the use of blood products by using more coagulation factors than fresh-frozen plasma [36]. As MT is practice-dependent, we presumed that MT is not a reliable outcome to assess the risk of bleeding.

### Clinical implication

Early identification of patients at risk of life-threatening bleeding is critical for the administration of life-saving interventions and for transport to the appropriate hospital. Scores using laboratory assays and imaging such as TASH are not useful because they cannot be used prehospital. The sensitivity of the scores’ predicting MT is too low for an appropriate use for prehospital triage. The inconclusive zone is too large to stratify the risk of life-threatening bleeding. These scores are able to discriminate only some high-risk patients probably when the bleeding is clinically obvious. More than three quarters of patients who died within 24 h were not identified by the SI and ABC scores. Moreover, predicting the receipt of particular types of medical care runs the risk of circularity with a high-risk of false prediction. The outcome of MT seems not appropriate as the performance of prehospital MT scores to predict early death is weak and could vary over the time. MT is not a patient centred outcome, is subject to practice changes and should not be used to stratify the risk of life-threatening bleeding.

The BATT score accurately predicts early death and facilitates the identification of patients with a low, intermediate, and high-risk of life-threatening bleeding. Because it can be used in the prehospital setting it is ideal for early decision-making. Early identification of non-obvious bleeding in the intermediate risk patient represents the most important intervention to avoid preventable death.

A BATT score ≥ 3 includes patients with an intermediate and high risk and has an undertriage of 5% and an overtriage of 50%. This overtriage rate considered acceptable by the American College of Surgeon [28] and so a BATT score ≥ 3 seems an appropriate cut off for triage. The high-risk BATT score (≥ 8) with less than 10% of overtriage may be useful for prehospital activation of MT protocol. There is recent evidence that many patients that could benefit from tranexamic acid treatment are not treated, in particular older women [37]. The use of the BATT score by paramedics could rationalize the use of tranexamic acid and help tackle inequalities (age and gender) [38].

## Conclusion

The BATT score accurately estimates the risk of early death with excellent performance, low undertriage and can be used for prehospital treatment decision-making. Although the TASH score presented good performance in-hospital, it is not suitable in prehospital. Scores predicting MT presented high undertriage. The outcome MT seems not appropriate to stratify the risk of life-threatening bleeding.

## Supplementary Information

**Additional file 1. 1**. Summary of development and validation method of each trauma bleeding score.**2**. Definition of statistics and performance indicators.**3**. Receiving Operating Curve (ROC) of bleeding scores for very early death within 12 h.**4**. Calibration Curves for external validation of BATT and TASH score.

## Data Availability

The datasets used during the current study belongs to the Swiss Trauma Registry (STR) and are available from the STR on reasonable request.
