# Supplementary material for: Early identification of bleeding in trauma patients: external validation of traumatic bleeding scores in the Swiss Trauma Registry
Source: Crit Care. 2022 Sep 28;26:296. doi: 10.1186/s13054-022-04178-8 (PMC9520811; doi:10.1186/s13054-022-04178-8)

**Supplementary files:**

**Supplement 1.** Summary of development and validation method of each trauma bleeding score.
**Supplement 2.** Definition of statistics and performance indicators
**Supplement 3.** Receiving Operating Curve (ROC) of bleeding scores for very early death within 12 hours
**Supplement 4.** Calibration Curves for external validation of BATT and TASH score

**Supplement 1. Summary of development and validation method of each trauma bleeding score.**

| | Development | Validation | External validation | | | | | |
|---|---|---|---|---|---|---|---|---|
| | | Design<br>Setting | Handle continuous variable | Outcome | Internal Validation | Handle Missing data | Setting | Handle Missing data |
| ABC | Based on local protocols and expert's opinion (16) | Retrospective.<br>Single center study (USA) from 2005 to 2006.<br>Inclusion: trauma team activation and receive blood (exclusion: death within 30 minutes) | Categories | MT (≥10 RBC the first 24h) as a surrogate of exsanguinating death from bleeding | Retrospective cohort (N=586) | No description | No external validation.<br>Temporal validation only (same setting at different time).<br>USA (N=1,018) (16) | No description |
| SI | Developed in 1967 as the ratio HR/SBP on 106 men with hypovolemia (Switzerland) (19) | Several studies in the Emergency department with critical illness patient.<br>In traumatic haemorrhage, validation in the German registry (N=21,853) (33), Northern French alps registry (N=3,689) (31) | Continuous | All-cause mortality, hemodynamic instability transfusion and massive transfusion | Retrospective cohort | Exclusion of observation with missing value. | Meta-analysis on trauma mortality and massive transfusion (37) | No description |
| TASH | Development of a prognostic model using multivariate regression model (N=4,527) (14) | Retrospective.<br>Multi-center study based on German trauma registry (Germany) | Categories | Massive transfusion (≥10 RBC the first 24h) Surrogate of severe bleeding | Split sample (no detail of the criteria of split) (N=1,517) (14) | Exclusion of observations with missing value. | No external validation.<br>Temporal validation only (same setting at different time).<br>German registry (N=5,834) (15) | Exclusion of observations with missing value. |
| BATT | Development of a prognostic model using multivariate regression model. (N= 23,430) (20) | Retrospective.<br>International multi-center study (41 countries; 287 trauma center) | Continuous and Polynomial terms | Death due to bleeding | Bootstrap, Internal-external validation. (N=22,422) (20) | Multiple imputation | UK trauma registry (N=104,862) (21) | Multiple imputation |

**Supplement 2.** Definition of statistics and performance indicators

## Brier Score

The Brier score is the quadratic score of the difference between the observed outcome and the predicted outcome.
Brier Score=$1/N$ x $\Sigma(Y-p)^2$
Which Y is the observed outcome and p the prediction of the model.

## Discrimination

Discrimination is the ability of the score to correctly identify patients with the outcome(19). Discrimination is assessed by sensitivity, specificity, Receiving Operating Characteristic (ROC) curve, likelihood ratio.

- **Sensitivity**

Sensitivity is the rate of true positive. The under-triage rate (false negative) is 1 minus the sensitivity.

- **Specificity**

Specificity is the rate of true negative. The over-triage rate (false positive) is 1 minus the Specificity.

- **ROC curve**

ROC curve is the sensitivity (true positives) on 1-specificity (false positives) for each score(35). An ideal score will reach the upper left corner (all true positive with no false positive) which correspond to an area under the ROC curve (AUROC) of 1.0. The concordance statistic (C-Statistic) for binary outcome corresponds to the AUROC. A C-statistic of 1.0 showed perfect discrimination ability.

- **Likelihood ratio**

The likelihood ratio is the likelihood of a positive score in a patient with the outcome compared to the likelihood of a positive score in a patient without the outcome(36). The positive likelihood ratio is the ratio of sensitivity to 1-specificity and the negative likelihood ratio is the ratio of 1-sensitivity to specificity. A positive likelihood ratio of 10 or above will result in a large increase in the probability of the outcome and a negative likelihood ratio of 0.1 or less will result in a large decrease in the probability of the outcome.

## Calibration

Calibration is the agreement between observed and predicted outcome.

- **Calibration in the large** is the difference between the mean predicted and observed probabilities.
- **The calibration ratio** is the ratio of the predicted and observed number of events (P/O).
- We plotted **calibration curve** as the observed and predicted probabilities of the outcome by decile of the score which corresponds to the Hosmer-Lemeshow goodness-of-fit test and with local regression based on LOESS algorithm(19). We estimated the calibration intercept and slope of the calibration plot as a measure of spread between predicted and observed outcome. Ideally, the intercept would be zero indicating that the predictions are neither systematically too low or too high and the slope would be 1.

**Supplement 3. Receiving Operating Curve (ROC) of bleeding scores for very early death**

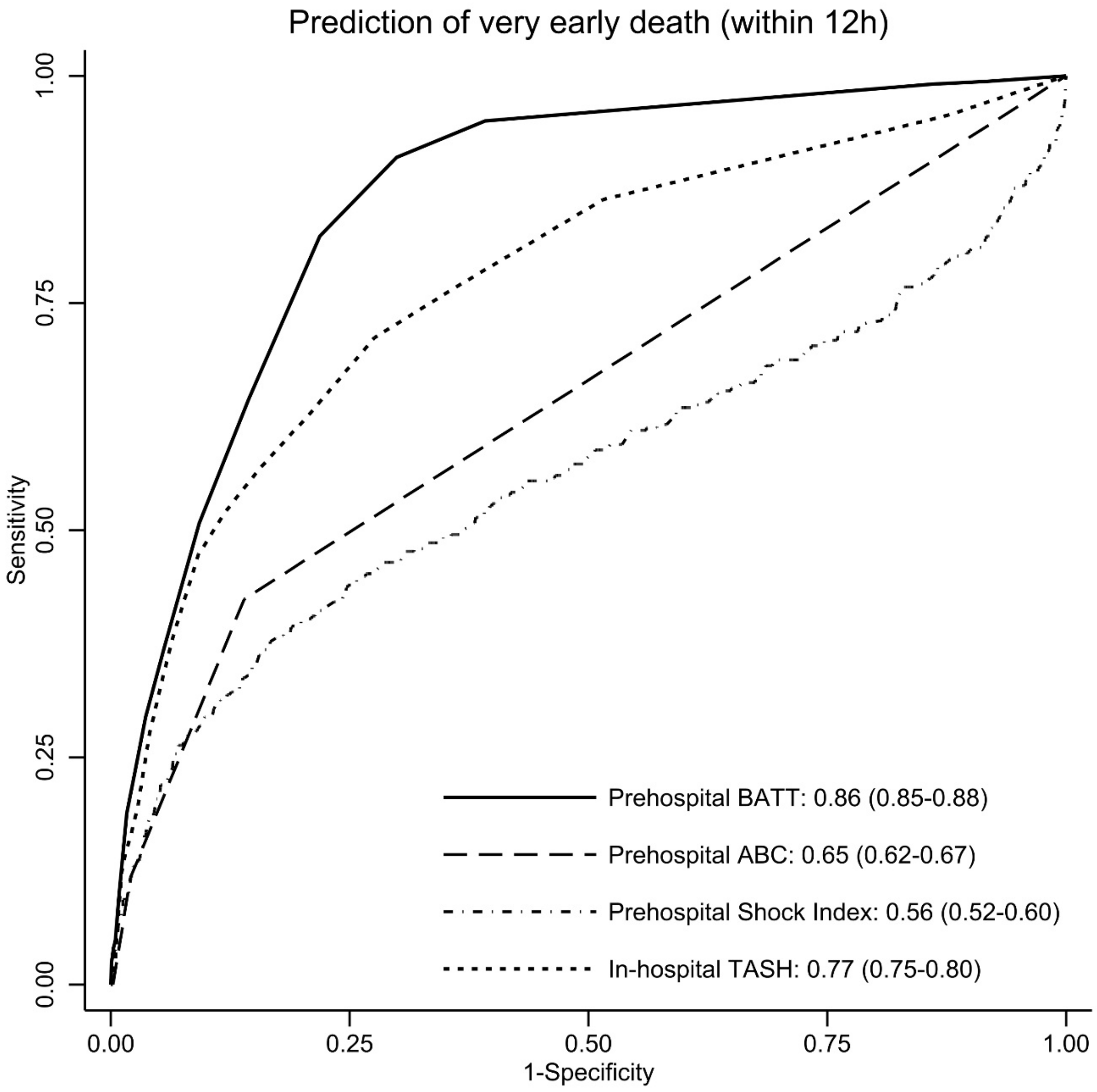

**Supplement 4. Calibration Curves for external validation of BATT and TASH score**

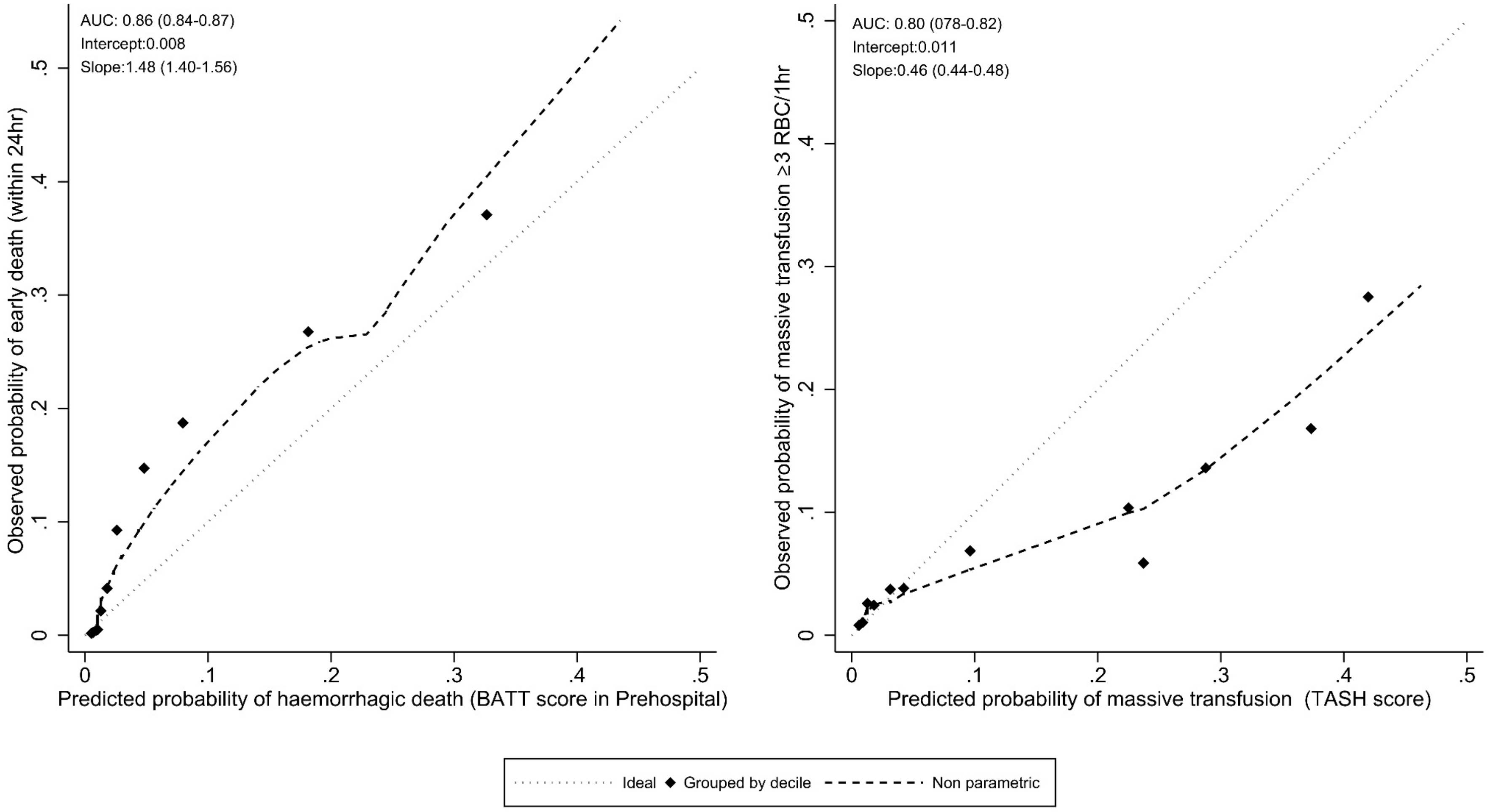

Supplement: Supplementary file 1 — Additional file 1. 1. Summary of development and validation method of each trauma bleeding score.2. Definition of statistics and performance indicators.3. Receiving Operating Curve (ROC) of bleeding scores for very early death within 12 h.4. Calibration Curves for external validation of BATT and TASH score. [file 13054_2022_4178_MOESM1_ESM.pdf]
